# Violence against women in the Philippines: barriers to seeking support

**DOI:** 10.1016/j.lanwpc.2022.100471

**Published:** 2022-05-03

**Authors:** Isabel Kristine M. Valdez, Ma.Veronica Pia N. Arevalo, Janine Patricia G. Robredo, Sabrina Laya S. Gacad, Marie Aubrey J. Villaceran, Gertrudes R. Libang, Edelina P. Dela Paz, Krissi Shaffina Twyla A. Rubin, Michelle Ann B. Eala

**Affiliations:** a College of Medicine, University of the Philippines, Manila, Philippines; b Ateneo School of Medicine and Public Health, Pasig City, Philippines; c Center for Women's and Gender Studies, University of the Philippines, Diliman, Quezon City, Philippines; d General Assembly Binding Women for Reforms, Integrity, Equality, Leadership, and Action (GABRIELA), Quezon City, Philippines; e Social Medicine Unit, College of Medicine, University of the Philippines, Manila, Philippines; f Center for Gender Equality & Women's Human Rights, Commission on Human Rights, Quezon City, Philippines; g College of Medicine, University of the Philippines, Manila, Philippines, 547 Pedro Gil Street, Manila, 1000, Philippines

The Philippines is among one of the most gender-equal countries in the Western Pacific region.​​^1^ Nevertheless, it is evident that the sociocultural landscape lags behind: one in four Filipino women has experienced gender-based violence, and 41% of victims do not seek help.^2^ Despite existing laws and a widespread local anti-violence against women (VAW) movement, multiple barriers to help-seeking exist, and it is ultimately the economic, sociopolitical and cultural structures in the Philippines hindering VAW victims from seeking support.

Like in other Asian countries, Filipino women are stifled by a patriarchal society emphasizing male dominance in family structures and larger social institutions.^3^ Traditionally, Filipino men are household heads and breadwinners; women are deemed subservient, hence economic abuse is common in VAW cases,^4^ and a high acceptance of justified wife beating exists.^2^ Women's pleasures are considered objects to pursue or control, hence they are regarded as a vulnerability. Few women seek help because of expectations to be self-sacrificing, thus giving up safety and security in favor of family reputation. Defying gender norms invites objectification, shame, guilt, and even justification of violence, hence the culture of victim-blaming.^3^

Through public debasing of women, condoning rape jokes and sexual remarks, openly harassing female supporters, associating femininity with weakness, and encouraging the military to “shoot women ‘communist rebels’ in the vagina,” the current administration under President Duterte personifies sexism, shaping society's perception of women. This misogyny is tolerated by many citizens, including some women of power. Coined “feminists of convenience,” these individuals advocate women's rights yet remain silent about the President's behavior for personal and family gains and to avoid political backlash.^3^ In their silence, the culture of impunity prevails.

It is apparent that women's rights is not the administration's priority, and this manifests systemically through complex referral pathways, fragmented documentation systems, and a slow judicial process. With stringent policies (curfews, checkpoints, and rationed quarantine passes) restricting mobility, this unsettling reality has intensified during the COVID-19 pandemic. VAW victims are trapped in their homes, unable to seek help and alternative shelter.^5^ Escalation of VAW-related and help-seeking internet search activity is not coincidental.^6^ Moreover, health, social, and legal services are largely inaccessible, a situation exacerbated by the diversion of national resources to the pandemic response. Reproductive health services are disrupted by 77-85%,^7^ and the adolescent birth rate is 31 per 1000 women. While 10.1% of all live births occur in the 15-19 age group, only 3.2% of these are sired by men of the same age,^8^ suggesting duress and power imbalance.^9^

Also vulnerable are women facing multiple and intersecting forms of discrimination, such as transgender women, indigenous women, women with disabilities, poverty-stricken women, and internally displaced women. The additional barriers of stigma, discrimination, State neglect, and harassment from law enforcers contribute to their distrust in the system, making them less likely to report to the police.^5^^,^^7^ Poverty and job insecurity aggravate the situation: women resort to prostitution, and online classes compound the risk of children's sexual exploitation with increased internet exposure.^5^^,^^11^

With the pandemic further threatening women's safety, the priority is ensuring functional, responsive, and accessible VAW responses that are survivor-centered and trauma-informed. Community-based first responders should still be reachable during quarantine. Healthcare providers must be trained for selective enquiry and first-line support of survivors.Referral pathways should be simplified to expedite care and assistance: upon identification of victims, a blanket referral to sexual and reproductive health providers, psychiatric aid, legal assistance, protective shelters, and livelihood assistance can be made. Establishing an active, centralized VAW surveillance system must take into consideration mobility under community restrictions. More accessible communication channels, like social media, must be made available and maximized. Marginalized women should be included in surveillance and protected in legislation, and VAW survivors should be consulted to improve service delivery.

Organizing women, educating them of their rights, promoting rights to pleasure and safety, and encouraging help-seeking behaviors while changing policies that increase vulnerability to VAW will foster women empowerment. Ensuring full implementation of the Magna Carta of Women^10^ is imperative in eliminating discrimination. This includes changing gender bias norms, non-discriminatory employment, leave benefits, equal opportunity for education and training, increased information access, and more women in leadership roles to advocate policy reform. Women prefer getting help from their community,^11^ hence community-based reporting and response systems should be strengthened in conjunction with bystander education to change sociocultural norms that condone VAW. Ultimately, institutional cultures perpetuating VAW must be tackled with interdisciplinary and intersectoral social and public health interventions, and the community must work hand-in-hand with an accountable government to end VAW in the Philippines.

## Contributors

IKMV and MVPNA were in charge of literature search, data analysis, interpretation, and writing. IKMV, MVPNA, JPGR, MABE, SLSG, MAJV, GRL, EDP, and KSTAR all worked to revise and review the manuscript.

## Declaration of interests

We declare no conflicts of interest.
